# Outcomes of a mandatory non-medical switch of infliximab to a biosimilar for inflammatory bowel disease in British Columbia, Canada

**DOI:** 10.1093/jcag/gwae011

**Published:** 2024-03-23

**Authors:** Thomas Tam Hoang, Jacqueline Reid, Cherry Galorport, Brian Bressler, Yvette Leung, Greg Rosenfeld

**Affiliations:** Department of Medicine, Faculty of Medicine, University of British Columbia, Vancouver, BC V5Z 1M9, Canada; Division of Gastroenterology, Department of Medicine, Faculty of Medicine, University of British Columbia, Vancouver, BC V5Z 1M9, Canada; Division of Gastroenterology, Department of Medicine, Faculty of Medicine, University of British Columbia, Vancouver, BC V5Z 1M9, Canada; Division of Gastroenterology, Department of Medicine, Faculty of Medicine, University of British Columbia, Vancouver, BC V5Z 1M9, Canada; Division of Gastroenterology, Department of Medicine, Faculty of Medicine, University of British Columbia, Vancouver, BC V5Z 1M9, Canada; Division of Gastroenterology, Department of Medicine, Faculty of Medicine, University of British Columbia, Vancouver, BC V5Z 1M9, Canada

**Keywords:** inflammatory bowel disease, infliximab, biosimilars

## Abstract

**Background:**

Despite infliximab biosimilars becoming widely used in inflammatory bowel disease (IBD) patients, real-world non-medical switching is sparse. A biosimilar non-medical switch was launched in British Columbia in 2019, the first Canadian province to do so, from Remicade to an approved biosimilar (CT-P13 or SB2).

**Aims:**

This study aims to obtain real-world evidence evaluating the clinical outcomes of non-medical switching from Remicade to the infliximab biosimilars.

**Methods:**

This is a retrospective observational study of stable IBD patients from the IBD Centre of BC who underwent the non-medical infliximab switch. The primary outcome is treatment continuation at 12 ± 2 months post-switch. Secondary outcomes include frequency of loss of response, adverse events, and immunogenicity within the first 12 months post-switch. A control group of patients maintained on the originator served as a comparison.

**Results:**

Patients in the biosimilar switch group (*n* = 264) and originator group (*n* = 99), show similar demographics and disease characteristics. There was no difference in infliximab continuation between the biosimilar group (94.9%) and the originator group (90.1%) (*P* = 0.18). Reasons for discontinuation of infliximab included loss of response (4.04% vs 4.91%), immunogenicity (1.01% vs 0.75%), or adverse effect (1.01% vs 2.3%) in the infliximab originator vs biosimilar switch group, respectively. Similarly, no differences in safety or efficacy were observed between patients switched to CT-P13 or SB2.

**Conclusions:**

Non-medical biosimilar switch of infliximab demonstrates similar clinical outcomes compared to originator molecule continuation for therapy of IBD. These data support the safety and efficacy of non-medical infliximab switching in IBD patients.

## Introduction

Infliximab is a monoclonal antibody that blocks tumour necrosis factor alpha (TNF-alpha), a key pro-inflammatory cytokine involved in chronic inflammatory diseases.^1^ It was initially approved in Canada under the trade name Remicade (Janssen) for use in Crohn’s disease (CD) in 2001, followed by ulcerative colitis (UC) in 2006.^2^ Remicade is currently used in adult and paediatric patients for moderate to severe UC and CD, as well as fistulizing CD.^1,2^

Biosimilars are biologic drugs that have a high degree of similarity but are not identical to their originator molecules.^3^ Biosimilars have been created as patents have expired for originator biologics, and although they require pharmacokinetic pre-approval testing, the clinical trial data is limited. This contributes to the manufacturer’s ability to offer the biosimilar at a lower price point.^3^ Commonly prescribed infliximab biosimilars for CD and UC in Canada include CT-P13 (Inflectra) and SB2 (Renflexis).^4^ The efficacy and safety of biosimilars have been studied extensively and they are generally accepted as a first-line therapy in biologic naïve patients, however, the data about biosimilar switching are more sparse.^4,5^

A joint position statement from the Canadian Association of Gastroenterology and Crohn’s Colitis Canada was published in 2020 comparing anti-TNF therapies with biosimilars.^4^ A recommendation was made against non-medical switching of originator infliximab to biosimilars in patients who are stable on therapy, although the recommendation is weak and based on the existing low-quality evidence.^4^

In 2019, a biosimilars initiative was launched in British Columbia, the first province in Canada to do so. This non-medical initiative was established to switch stable patients from the originator drug (Remicade) to a Health Canada approved biosimilar (CT-P13 or SB2) because of the economic benefit to the BC Health Care system. The switch was mandatory, meaning that all publicly funded Remicade patients would need to switch to a biosimilar. For IBD patients, the switch period occurred from September 5, 2019 to March 5, 2020.

This study aims to obtain real-world evidence evaluating the clinical outcomes of this non-medical switch from Remicade to the infliximab biosimilars CT-P13 or SB2. Specifically, we look at frequency of treatment persistence, adverse effects, loss of response, and immunogenicity following the mandated switch.

## Methods

### Study design

This is a single-centre retrospective observational study conducted at the IBD Centre of BC, a tertiary referral centre for IBD patients affiliated with St. Paul’s Hospital and the University of British Columbia in Vancouver, Canada. Ethics approval was obtained from the local ethics review board. Patients eligible for the switch from Remicade to a biosimilar were identified by a search of the IBD Centre’s electronic medical record between January 1, 2006 and October 1, 2021. Patients were categorized into a “biosimilar group” defined as those who were switched from Remicade to CT-P13 or SB2, or a control “originator” group of patients who remained on Remicade throughout the switch period. Only patients receiving publicly funded Remicade were made to switch and therefore, patients who did not meet these criteria remained on Remicade throughout the study period and formed the control group. Detailed data abstraction was then performed by J.R and T.T.H.

Inclusion criteria included adults 18 years or older with a diagnosis of CD or UC clinically, endoscopically, and/or radiologically. Patients must have been previously receiving maintenance therapy with Remicade and subsequently switched to a biosimilar during the mandated switch period of September 5, 2019 to March 5, 2020. Patients must have been actively receiving Remicade at the time of the switch, defined as those who have received at least 2 infusions of the biosimilar at the usual maintenance dosing interval for infliximab.

Exclusion criteria included inadequate follow-up to determine biosimilar continuation, patients switched due to clinical deterioration on Remicade, or patients who received the biosimilar more than 7 days later than anticipated based on Remicade dosing interval prior to switching. Biologic-naïve patients who underwent induction therapy with CT-P13 or SB2 were not included in this study.

Data collected included basic demographics, concurrent immunomodulator use with azathioprine or methotrexate, duration on Remicade prior to the switch date, pre-switch C-reactive protein (CRP), or faecal calprotectin (FCP), and disease extent by Montreal Classification. Post-switch clinical outcomes documented included remainder duration to infliximab, immunogenicity, disease complications, and reasons for discontinuation.

### Study outcomes

The primary outcome is infliximab treatment continuation at 12 ± 2 months post-switch for the biosimilar group. The control group was assessed 12 ± 2 months following December 2019 (the mid-point of the switch period). Infliximab continuation was defined as ongoing biologic infusions 12 months post-switch, as noted in infusion reports or other clinical documentation. Secondary outcomes included reasons for discontinuation such as loss of response, adverse events while on infliximab, or patient factors (defined as a patient-directed decision to discontinue infliximab for reasons not listed above). Loss of response was defined as worsening of disease either clinically or endoscopically while receiving regular infliximab therapy following the switch, and any subsequent rescue treatments were also documented. Dosage changes, therapeutic drug monitoring levels, and immunogenicity (loss of response secondary to undetectable drug levels in the presence of anti-infliximab antibodies) were recorded when available.

### Statistical analysis

Categorical variables were expressed as proportions (%) or median values ± interquartile range (IQR) and analyzed using Pearson’s *X*^2^ or Fisher’s exact test where applicable. Quantitative variables were expressed as means ± SD and analyzed via Mann–Whitney *U* test. Statistical analysis was performed using SPSS Statistics version 29 for Mac. *P*-values < 0.05 were deemed significant. Kaplan–Meier survival analysis was performed to demonstrate treatment continuation with stratified log-rank (Mantel–Cox) for between-group comparisons. Propensity score matching (PSM) with Cox regression analysis via proportional hazards model was also performed to adjust for potential confounders (pre-switch Remicade duration, age at diagnosis, and concurrent immunomodulator use).

## Results

### Study demographics

**Figure 1.**
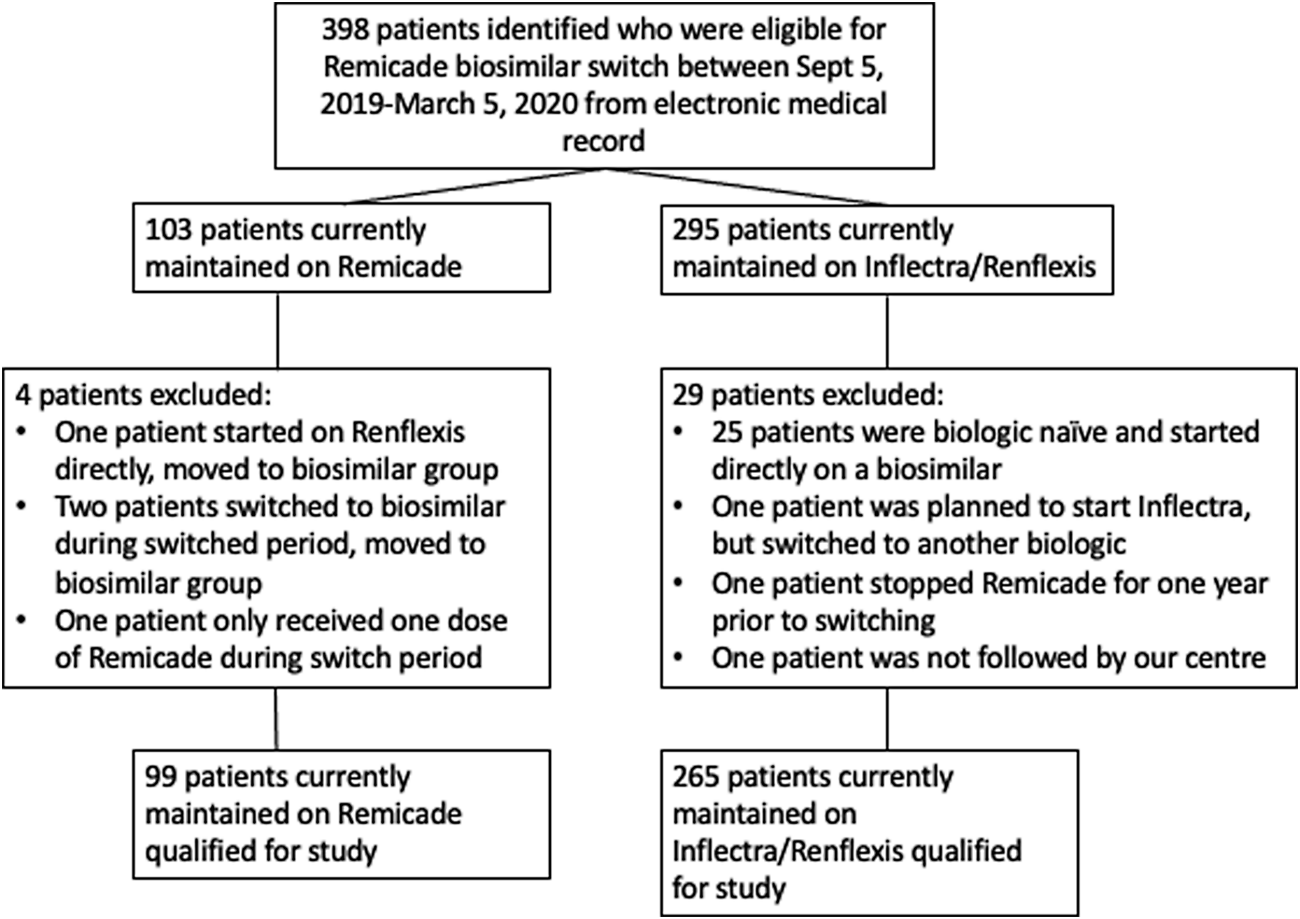
Flow diagram depicting patient selection with relevant inclusion/exclusion criteria. No patients were lost to follow-up by the study endpoint of September 1, 2021.

In total, 364 patients who met inclusion criteria were identified. Of those, 265 patients underwent a non-medical biosimilar switch during the mandated period of September 5, 2019 to March 5, 2020, while 99 patients remained on the originator Remicade, either through compassionate or private coverage during this same period (Figure 1). Patient demographics are summarized in Table 1, demonstrating similar clinical baselines between both cohorts. No statistically significant differences were observed with respect to disease characteristics between both groups, with the exception of the switch group being slightly younger at the time of diagnosis (26.2 ± 10.7 vs 23.4 ± 10.7 years, *P* = 0.012). Approximately two-thirds of total patients had CD (*n* = 247) and one-third had UC (*n* = 117), which is reflected in both cohorts and consistent with the background IBD population at our centre (data not shown). Approximately 41% of the 265 switchers were continued on CT-P13, while the remainder transitioned to SB2. Patients who remained on the originator molecule had a shorter mean total duration on Remicade before the switch (69.5 ± 42.7 months) compared to patients who switched to a biosimilar (93.2 ± 39.9 months) (*P* <0.01).

**Table 1.** Baseline patient characteristics are similar for both the originator and switch cohorts.

	Infliximab originator	Infliximab biosimilar switch	*P*-value	Infliximab biosimilar switch (propensity score matched)	*P*-value	Test
*N*	99	265		99		
Gender (M)	56 (54.5)	153 (57.7)	0.64	52 (52.5)	0.89	*χ* ^2^
Age (years)	38.2 ± 11.8	39.1 ± 13.0	0.38	38.6 ± 12.0	0.81	*t* test
Age at IBD diagnosis (years)	26.2 ± 10.7	23.4 ± 10.7	0.012	24.7 ± 10.0	0.31	*t* test
Crohn’s	65 (65.6)	182 (68.9)	0.62	72(72.7)	0.36	*χ* ^2^
Age at diagnosis	N (%)	N (%)	0.01		0.13	*χ* ^2^
A1 (<16)	7 (10.7)	54 (29.7)		17 (23.6)		
A2 (17–40)	54 (83.1)	118 (64.8)		50 (69.4)		
A3 (>40)	4 (6.15)	10 (54.9)		5 (6.94)		
Location			0.21		0.20	*χ* ^2^
L1 (ileal)	17 (26.1)	48 (26.4)		19 (26.4)		
L2 (colonic)	21 (32.3)	48 (26.4)		18 (25.0)		
L3 (ileocolonic)	27 (41.5)	84 (46.2)		33 (45.8)		
L4 (isolated upper tract disease)	0 (0)	3 (1.6)		2 (2.78)		
Behaviour			0.387		0.46	*χ* ^2^
B1 (non-stricturing, non-penetrating)	40 (61.5)	102 (56.0)		44 (61.1)		
B2 (stricturing)	9 (13.8)	44 (24.1)		17 (23.6)		
B3 (penetrating)	16 (24.6)	35 (19.2)		11 (15.3)		
Perianal disease modifier	35 (53.8)	86 (47.2)		29 (40.3)	0.13	*χ* ^2^
UC	34 (34.3)	83 (31.3)	0.62	61 (61.6)	0.04	*χ* ^2^
Ulcerative proctitis	0 (0)	7 (8.4)		1 (2.70)		
Left-sided colitis	11 (32.3)	39 (47.0)		16 (43.2)		
Extensive colitis	23 (67.6)	37 (44.6)		10 (27.0)		
Concurrent immunomodulator therapy (azathioprine/methotrexate)	27 (27.2)	19.2 (51)	0.11	32 (32.3)	0.75	*χ* ^2^
Duration on Remicade prior to switch	69.5 ± 42.7	93.2 ± 39.9	<0.001	82.0 ± 34.8	0.026	*t*-test
Pre-switch CRP (mg/L)	3.65 ± 5.97	2.89 ± 10.1	0.54	1.98 ± 2.86	0.029	*t*-test
Pre-switch FCP (mcg/g)	808 ± 1704	199.55 ± 449	0.22	180 ± 502	0.041	*t*-test

Biosimilar switch patients had a longer mean duration on Remicade pre-switch compared to the originator cohort (*P* < 0.001). This difference remained despite PSM to control for this potential confounding (*P* = 0.026). Pre-switch CRP/FCP were not available for 25.2%/64 of the originators and 8%/50% of the switchers. While there is a difference post-PSM, the average time between pre-switch CRP/FCP and date of switch was 8.8 ± 11/13.6 ± 16.0 months for the control and 12.5 ± 19.8/21.0 ± 22.0 for the unadjusted switch group respectively. Proportions reported as *N*(%), means as mean ± SD.

### Treatment persistence

**Figure 2.**
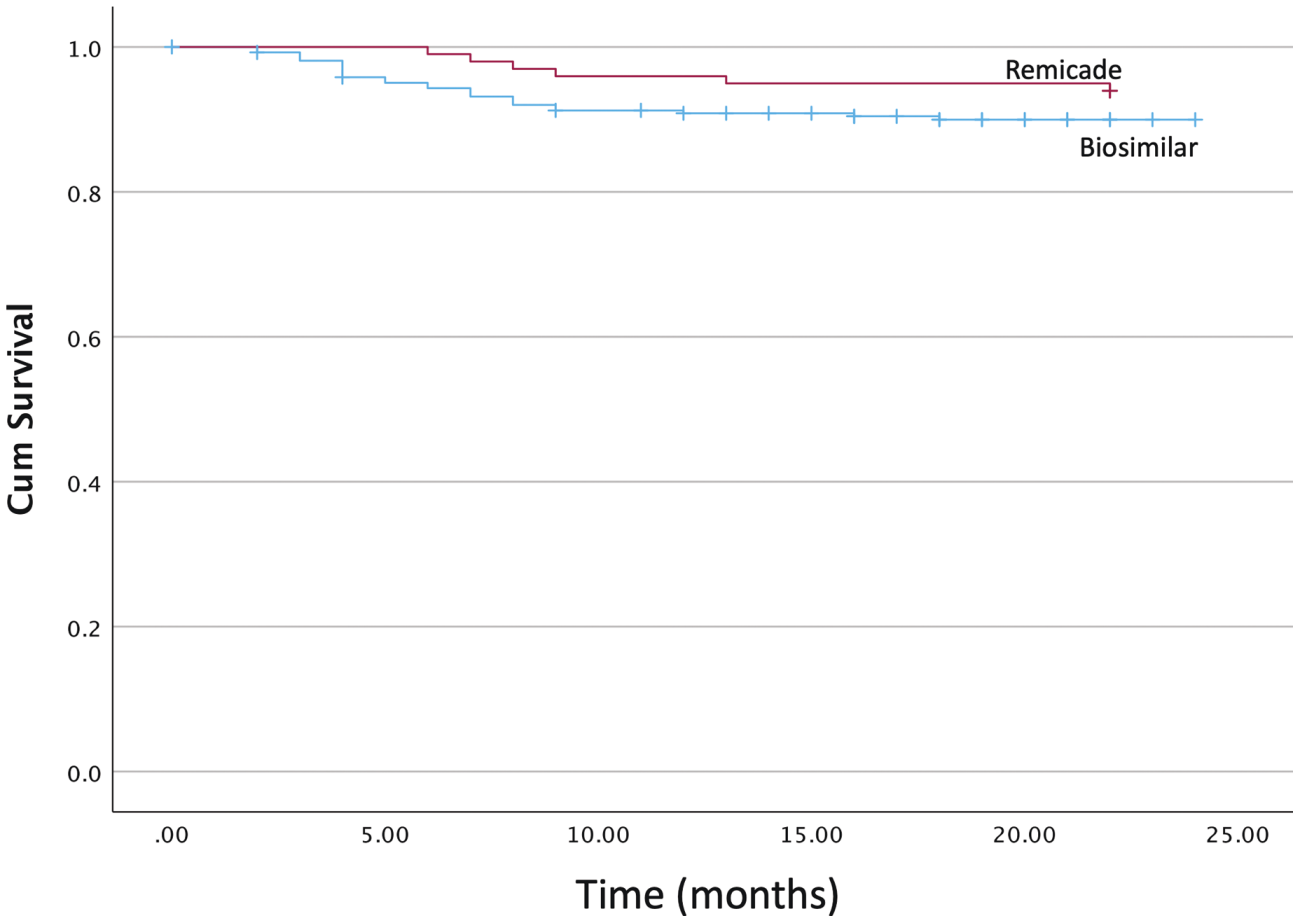
Kaplan–Meier survival analysis shows no difference in all-cause treatment discontinuation was observed between the Remicade originator and biosimilar switch groups (log-rank *P* = 0.175).

**Figure 3.**
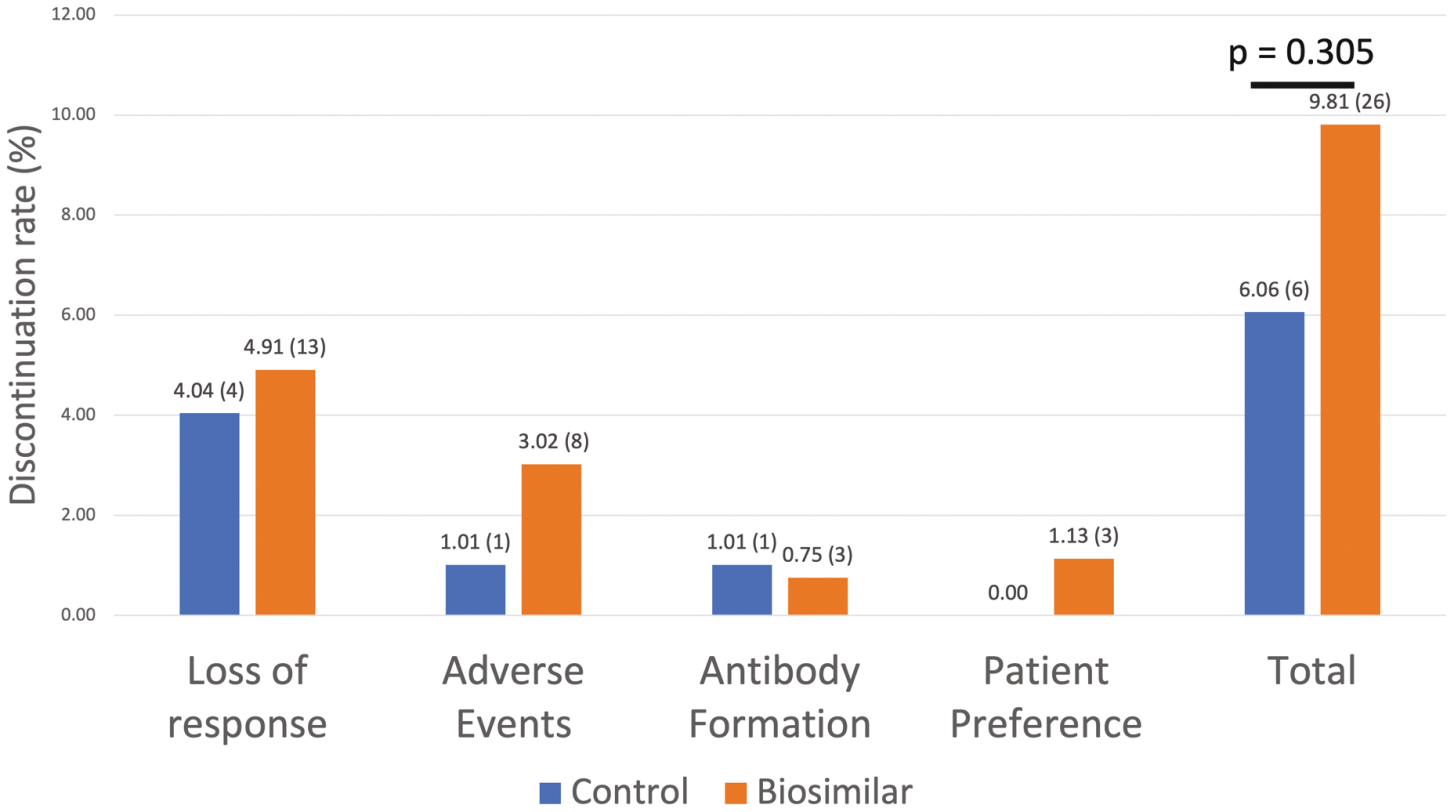
Rates of infliximab discontinuation by 12 months post-biosimilar switch between infliximab originator group (*n* = 99) and biosimilar switch group (*n* = 265). *N* values indicated in parentheses.

The endpoint of data collection was September 1, 2021. The mean duration of continuation on a biosimilar following the switch was 18 ± 5.15 months, though clinical outcomes were documented during the first 12 ± 2 months of follow-up. While there is a numerical difference, there is no statistically significant difference (*P* = 0.18) in the frequency of infliximab treatment continuation by the first 12 months following the switch (239, 90.1%) compared to the Remicade originator control group (93, 94.9%), with Kaplan–Meier survival analysis demonstrating similar rates of treatment persistence following switching from Remicade to the biosimilars (Figure 2). Of the 90.1% of biosimilar patients who remained on infliximab at 1 year, 167 (69.9%) and 72 (30.1%) had CD and UC, respectively (91.7% of CD and 86.7% of UC patients in the switch cohort). In comparison, the 94.9% of patients who continued treatment in the control group were comprised of 62 (66.7%) CD and 31 (33.3%) UC patients (95.3% and 91.2% of control group CD and UC patients, respectively). All causes of treatment discontinuation are summarized in Figure 3, again showing no significant differences following the biosimilar switch (9.81% biosimilars vs 6.06% originators, *P* = 0.305). A subgroup analysis individually comparing Remicade, CT-P13, and SB2 also showed no statistically significant difference in the all-cause treatment discontinuation rate (Figure S1) with similar treatment persistence (Figure S2, *P* = 0.29). PSM was performed to control for potential confounding by immunomodulator use, pre-switch duration on Remicade, and age of IBD diagnosis (Table 1). Subsequent Cox regression via proportional hazards model demonstrated that while switchers were approximately twice as likely to experience treatment discontinuation, this difference was not statistically significant (hazard ratio = 2.11 (CI: 0.57–1.04, *P* = 0.148)). Subgroup analysis by IBD subtype on the PSM population also demonstrated no statistically significant difference for CD (hazard ratio = 3.39 (CI = 0.88–13.0), *P* = 0.08) and UC (hazard ratio = 0.863 (CI = 0.13–5.17, *P*= 0.14)).

### Loss of response

In the biosimilar group, 13 patients (6 CD and 7 UC) stopped treatment due to a loss of response. Endoscopic evidence of newly active disease was observed in 5 patients. The remaining 7 were diagnosed clinically with worsening symptoms and elevated inflammatory markers (CRP and FCP). One patient required hospitalization and systemic steroids due to their disease flare. Disease control was successfully achieved again in all patients following a second switch to vedolizumab (9), adalimumab (2), and ustekinumab (2). In comparison, 4 patients (2 CD and 2 UC) stopped treatment due to a loss of clinical response in the originator cohort (RR = 1.21, 95% CI = 0.38–23.6, *P* = 0.30). Two patients achieved disease control again after switching to vedolizumab, while the remaining 2 developed refractory disease requiring total colectomies. Patient clinical outcomes following loss of response are summarized in Table S1.

### Adverse events

The frequency of adverse events is summarized in Table 2. Eight patients (6 CD, 2 UC) in the biosimilar cohort were discontinued following an adverse reaction on infliximab. This included 2 cases of new-onset polyarthralgias that improved following a switch to another biosimilar and reverting to Remicade, as well as one case each of facial rash, allergic-type reaction with facial angioedema, self-limiting diarrhoea post-infusion, drug-induced lupus, and drug-induced pulmonary nodules, all of which were medically managed. There was one additional reported case of a breast angiosarcoma requiring partial mastectomy. In comparison, one adverse event was reported in the originator cohort, with one case of infliximab-induced vasculitis in a CD patient requiring treatment discontinuation and medical management. While there is a numerical increase in adverse events in the switch cohort, this difference is not statistically significant (RR = 2.98, 95% CI = 0.38–23.6, *P* = 0.30). Three switch cohort patients discontinued infliximab secondary to unrelated factors including pregnancy, undisclosed personal reasons, and the COVID-19 pandemic, respectively. Table 3 depicts the frequency of adverse effects between the control and the biosimilar groups subdivided into SB2 and CT-P13, again demonstrating no significant difference in clinical outcomes following the switch period.

**Table 2.** Outcomes of the biosimilar switch demonstrate a numerical but not statistically significant increase in the rate of infliximab discontinuation in the switch cohort (*P* = 0.305)..

	Infliximab originator	Infliximab biosimilar switch	*P*	Test
Duration on biosimilar after switch	–	18.0 ± 5.15		
Discontinuation of infliximab	6 (6.06)	26 (9.81)	0.305	*χ* ^2^
Reason for discontinuation				
Loss of response	4 (4.04)	13 (4.91)		
Antibody development	1 (1.01)	2 (0.75)		
Adverse effect	1 (1.01)*	8 (3.02)**		
Other patient factors	0	3(1.13)***		

Proportions reported as *N*(%), means as mean ± SD.

*Drug-induced vasculitis × 1, **arthralgias × 2, allergic reaction × 1, drug induced lupus × 1, rash × 1, drug-induced pulmonary nodules × 1, new angiosarcoma, self-limiting diarrhoea post-infusion, ***pregnancy × 1, COVID pandemic × 1, patient elected to stop × 1.

**Table 3.** A sub-analysis of biosimilar switch outcomes between Remicade and SB2/CT-P13 individually shows no statistically significant difference in rates of treatment discontinuation.

	Remicade (*n* = 99)	SB2 (*n* = 109)	CT-P13 (*n* = 156)	*P*	Test
Duration on biosimilar after switch	–	18.4 ± 4.80	17.6 ± 5.36	0.19	*t*-test
Discontinuation of infliximab	6 (6.06)	9 (8.3)	17 (10.9)	0.534	*χ* ^2^
Reason for discontinuation					
Loss of response	4 (4.04)	6 (5.50)	7 (4.49)		
Antibody development	1 (1.01)	0	2 (128)		
Adverse effect	2 (1.01) (infliximab-induced vasculitis)	2 (1.83) (rash × 1, allergic reaction × 1)	6 (2.56) (arthralgias × 2, drug-induced lupus × 1, drug-induced pulmonary nodules × 1, self-limiting diarrhoea post-infusion × 1, new angiosarcoma × 1		
Other patient factors	0	1 (0.92) (COVID)	2 (2.56) (pregnancy × 1, patient elected to stop × 1)		

Proportions reported as *N*(%), means as mean ± SD.

### Drug persistence

Drug level monitoring data was available for 61/265 biosimilar switch and 15/99 control patients during the first 12 months following the mandated switch period. Of the 61 comparator patients, 6 (9.8%) demonstrated subtherapeutic levels, of which 2 (3.28%) resulted in drug cessation following loss of response with detection of anti-infliximab antibodies. The remaining four patients reached therapeutic drug levels following dose escalation and remained on the biosimilar. In comparison, 15 control patients had available data, for which only one (6.67%) demonstrated subtherapeutic drug levels following a loss of response where anti-infliximab antibodies were detected, leading to drug cessation. Another had subtherapeutic levels which improved following a dose adjustment and thus continued infliximab. The remainder showed therapeutic or supratherapeutic levels.

## Discussion

In this retrospective observational study using real-world Canadian data, we demonstrate that switching from the Infliximab originator Remicade to biosimilars CT-P13 or SB2 is not associated with any significant adverse clinical outcomes for IBD patients on maintenance biologic therapy. Specifically, we demonstrate no statistically significant differences in rates of treatment persistence, loss of response, adverse events, or immunogenicity by one year of switching.

Our results are largely in keeping with the NOR-SWITCH Trial, the first randomized-control study investigating biosimilar switching for IBD, rheumatologic, and dermatologic conditions, demonstrating non-inferiority of CT-P13 compared to Remicade for all conditions. The authors concluded that no significant differences in disease exacerbation or adverse events were observed.^6^ However, in further IBD subgroup analysis of the NOR-SWITCH extension trial, stable patients on Remicade were compared to those on infliximab maintenance switched to CT-P13 with disease worsening occurring in 20.6% vs 13.1% of CD patients (risk difference 7.9%, 95% CI = 5.2–21) and 15.4% vs 2.9% of UC patients (risk difference 12.4%, 95% CI = 0.1–25).^6^ Although the authors reported similar efficacy, safety, and immunogenicity in both study arms, non-inferiority could not be concluded due to low patient numbers. Six other observational studies have since directly compared Remicade continuation to biosimilar switching which, while showing heterogeneous results, overall support the safety and efficacy of biosimilar switching.^7–12^ To the best of our knowledge, we are the first study to report comparator observational data from clinical practice in Canada.

Our reported 9.81% and 6.06% frequencies of all-cause treatment discontinuation in the switch and control cohorts, respectively, are lower than some previous studies that have reported post-switch biosimilar discontinuation rates as high as 21.7% for a similar follow-up duration, which the authors attributed to a possible nocebo effect.^12^ This phenomenon, where patients with negative expectations about treatments become more likely to experience a negative outcome, has been cited by other studies that have also reported a slightly higher treatment discontinuation rate among switchers.^13^ While no prominent effect was observed in our study, we do note a numerical but non-statistically significant increase in adverse events in the switch group, and that all 3 patients who requested treatment cessation for non-medical reasons were switchers. Another unique aspect of our cohort is that our centre employs clinical IBD nurses who assisted in counselling patients regarding the switch, which may have mitigated the nocebo effect previously reported. This effective strategy was previously documented by Armuzzi et al. to facilitate transitioning patients onto biosimilars.^14^ We also report an adverse event rate leading to treatment cessation of 1.01% and 3.02% in the control and switch group respectively, which is marginally lower but comparable to the 3%–4% reported in the NOR-SWITCH trial and approximate 4.5% aggregate frequency reported from multiple observational studies and a meta-analysis.^5,7,8,15–20^ While we demonstrate a trending increase in the rate of adverse effects in the switch group, our limited sample size does not allow us to conclude a statistically significant difference. Similarly, the rates of treatment cessation due to loss of response of 4.0% in the control and 4.9% in the switch group are comparable to similar rates reported at a median of one-year post-switch by Haifer et al., who demonstrated 7% and 8%, respectively.^9^ For the 15% and 23% of control and switch group patients where drug monitoring data was available, we also did not document any significant difference in subtherapeutic drug levels secondary to immunogenicity which is also in keeping with previous reports (^18–20^).

Our study does contain several limitations. First, due to the unavailability of retrospective data, we were unable to objectively determine remission at the time of biosimilar switching. As per the mandated provincial switch program, patients were determined by physician judgement to be clinically stable in the outpatient setting prior to switching, and therefore, no patients were in a symptomatic disease flare at the time of switch. While PSM was used to control for several confounders including immunomodulator use and pre-switch Remicade duration, we were unable to control for objective disease activity, serum inflammatory markers, and previous drug immunogenicity. It is possible some patients may have been experiencing mild symptoms or had subclinical disease activity at the time of switch, which is the nature of real-world data as opposed to clinical trial data. Our study was also underpowered to comment on the observed numerical difference in adverse event rates. Second, we could not reliably compare serum drug persistence and immunogenicity due to the lack of retrospective data available in our cohort, and the small proportion of patients with therapeutic drug monitoring available limits our analyses.

Given the increasing prevalence of non-medical switching initiatives across new jurisdictions, our study contributes real-world clinical experience from a mandated non-medical switch in Canada.^4^ These results support the existing clinical data on infliximab biosimilar switching from Europe and Asia, which can inform future practice guidelines and reassure healthcare providers about the safety and efficacy of biosimilar switching in IBD.

## Supplementary data

Supplementary data are available at *Journal of the Canadian Association of Gastroenterology* online.

gwae011_suppl_Supplementary_Materials

## Data Availability

The data underlying this article are available in the article and in its online Supplementary Material.
