# Supplementary material for: Outcomes of a mandatory non-medical switch of infliximab to a biosimilar for inflammatory bowel disease in British Columbia, Canada
Source: J Can Assoc Gastroenterol. 2024 Mar 23;7(4):299–305. doi: 10.1093/jcag/gwae011 (PMC11317628; doi:10.1093/jcag/gwae011)
Supplement: gwae011_suppl_Supplementary_Materials [file gwae011_suppl_supplementary_materials.zip › gwae011_suppl_Supplementary.docx]

**Table S1:** Clinical descriptions of losses of response following biosimilar switch vs. Remicade controls.

**Figure S1** – Rate of infliximab discontinuation between Remicade (n=99), (CT-P13 (n=156) and SB2 (n=109) at 12 months following biosimilar switch. N values are in parentheses.

**Figure S2**: No difference in all-cause treatment discontinuation between CT-P13 and SB2 (log-rank p=0.29).
